# Quantifying tracking quality during occlusion with an integrated gaze metric anchored to task performance

**DOI:** 10.1038/s41598-025-17519-8

**Published:** 2025-08-29

**Authors:** Tuisku Tammi, Jami Pekkanen, Benjamin Ultan Cowley, Otto Lappi

**Affiliations:** 1 Cognitive Science, University of Helsinki, Helsinki, Finland; 2 Faculty of Educational Sciences, University of Helsinki, Helsinki, Finland

**Keywords:** Human behaviour, Psychology, Perception, Visual system

## Abstract

Visual tracking of moving objects, even when they are temporarily hidden from view, is a fundamental skill for humans. This study examines gaze behavior in an object tracking task with intermittent occlusions, highlighting the role of task goals. Participants tracked an object moving along a linear path with random occlusions, performing a visual discrimination task upon the object’s brief reappearance. We analyzed gaze behavior and developed a model to quantify the relationship between gaze dynamics and task performance. The model yields a performance metric that takes into account the full gaze signal, i.e. both smooth pursuit and saccades. We introduce a position- and velocity-integrated tracking quality metric based on these gaze dynamics, providing an objective continuous measure of tracking performance that works also throughout occlusions.

**Supplementary Information:**

The online version contains supplementary material available at 10.1038/s41598-025-17519-8.

## Introduction

Humans are very effective in tracking visual targets, even when those targets become temporarily hidden from view. In everyday life, we routinely keep an eye on a single child in a busy playground or watch out for approaching cars before crossing the road. Similarly, in many skilled domains—such as sports or driving—efficient object tracking supports high-performance visuomotor behavior. Understanding the principles of the tracking system can thus shed light on an important component of everyday and expert skill. But what does ‘good’ or ‘effective’ tracking actually look like?

Intuitively, the idea of optimal tracking appears trivial: gaze should stay on the target (zero position error), and its velocity should match that of the target (zero retinal slip, or unity gain). This conception is reflected in common analysis practices, where the gaze signal is de-saccaded and pursuit velocity is then compared to target velocity, with unity gain representing presumed ideal tracking^1,2^. However, it has long been known that visual tracking relies on an interplay of smooth pursuit and saccades^3,4^: pursuit approximates but rarely exactly matches target velocity, while saccades rapidly reposition gaze to catch up or jump ahead. Especially during occlusion, saccades and pursuits work in tandem: smooth pursuit continues after object disappearance, albeit typically with a gradual decrease in velocity^5–7^, and is often accompanied by both compensatory and anticipatory saccades^8^.

This interplay may seem paradoxical. Humans are very good at demanding visual tracking tasks, yet tracking behavior may appear noisy and fragmented, with frequent saccades. Should smooth pursuit be considered the ideal strategy and saccades as noisy deviations or suboptimal compensation, rather than an integral part of tracking? To some extent, this assumption seems to rest on a conceptual conflation of ’tracking’ and ’pursuit’. The widespread use of de-saccaded pursuit gain as an indicator of tracking quality may inadvertently limit our understanding of how smooth and rapid eye movements contribute to object tracking, particularly under uncertainty (for example, occlusion).

In this study, we revisit the idea that minimizing position and velocity errors—that is, achieving smooth pursuit with near-unity gain—is inherently ‘optimal’. We argue that the effectiveness of gaze behavior in visual tracking should be evaluated based on careful empirical consideration of task conditions, objectives and measured performance in the specific task at hand; for example, how gaze behavior supports task outcomes such as target interception or discrimination accuracy, instead of ’pursuit performance’. Therefore, we explore the full gaze dynamics of tracking, including both pursuit and saccades, and link them to specific performance goals of the overall task. We apply this approach to a simple but well-studied tracking paradigm: a ramp stimulus with occlusion^9^. Moreover, we provide an objective tracking quality metric that is anchored to behavioral task performance, computed on the full gaze signal, and incorporating both relative position and velocity information.

Generally, tracking the object by smooth pursuit with unity gain is (implicitly or explicitly) assumed to be the ideal strategy^7^, as it stabilizes the object on the retina, minimizes velocity and positional errors, and aids in motion prediction^10^. However, this assumption can overlook the importance of saccades, which are essential, for example, to minimize positional error in tasks requiring fine visual discrimination. This is not necessarily because the pursuit system is incapable of generating perfect-gain pursuit, but because a saccadic strategy may better align with the specific objectives of the task^11,12^. Moreover, gaze behavior can adapt flexibly based on the participant’s prior experiences and expectations^7,13–15^. The ’desired’ gaze behavior thus depends on the task design and instructions given to participants^7^: for example, in tasks primarily requiring velocity estimation, smooth pursuit has been shown to improve performance^16,17^, while in tasks requiring high spatial accuracy, saccades are used to rapidly (re-)foveate the target^11,18^. In prior research on occluded tracking, a variety of instructions or tasks have been provided to participants to encourage tracking when the target is not visible. These include estimating time-to-contact^19^, estimating or discriminating reappearance location^16,20,21^, manual interception^22^, or simply maintaining tracking of the invisible target^5,9,23^.

In our study, participants track an object moving along a linear horizontal trajectory with random-duration occlusions, each occlusion period concluded with a visual discrimination task. Importantly, the visual discrimination target reappears only briefly after the occlusion. Because accurate visual discrimination requires having the target in (para)foveal vision, our task design requires participants to track the target sufficiently accurately throughout the occlusion—in both positional and velocity terms—to enhance the probability of successfully identifying the target upon reappearance. Here, we provide a detailed characterization of gaze behavior across different phases of the task (anticipation, visually guided tracking, and occluded tracking). Even in this relatively simple and frequently-studied design of linear tracking with occlusion, we observe intricate patterns in gaze behavior. Such detailed understanding is important for parameterizing gaze behavior and developing performance metrics that objectively assess tracking quality.

Based on our task design and thorough characterization of tracking behavior, we construct a tracking quality metric. Discrimination task performance provides an empirical benchmark for this measure, removing the need to assume, for example, unity gain of a de-saccaded gaze signal as an a priori criterion. Rather than isolating one type of eye movement, our approach includes the full gaze signal. We integrate two measures of visual stabilization: displacement (gaze-target positional difference) and slippage (gaze-target velocity difference). This integrated tracking quality metric allows for an assessment of tracking performance during occlusion.

## Methods

### Participants

Ten participants (8 females, 2 males, aged between 21 and 40 years) were recruited from university mailing lists. The participants reported normal or corrected-to-normal visual acuity and no known conditions that affected eye movements. One participant was excluded from analyses due to a low overall success rate in the discrimination task (38 %). Participants were remunerated with activity vouchers for their participation.

### Ethics

The study was conducted in full compliance with the ethical guidelines of the Finnish National Board on Research Integrity (TENK) and the University of Helsinki Research Ethics Committee in the Humanities and Social and Behavioural Sciences. According to the guidelines in effect at the time of our experiment, ethical review was not required because our study did not include any of the criteria for review. All participants provided written informed consent and were free to revoke their participation at any point.

### Materials

Eye movements were recorded with a binocular, head-mounted Pupil Core eye-tracker^24^, with the associated open-source Pupil Capture software v0.9.12 (https://github.com/pupil-labs/pupil) used for recording and calibration. The eye cameras recorded at 120 Hz with a resolution of 640$\times$480 pixels, while the forward-facing scene camera recorded at 60 Hz with a resolution of 1280×720 pixels. Four optical markers were placed on screen corners to allow for head pose estimation to map gaze from the headset’s forward-facing scene camera image to screen coordinates.

The experiment was presented on a LG OLED55C7V 55” screen with participants sitting at a fixed distance of approximately 85 cm on a Playseat Evolution gaming chair (Playseat Evolution Alcantara, Playseats B.V., the Netherlands). The software for the tracking task is available open-source at (https://github.com/jampekka/webtrajsim/tree/speedest18). All software ran on an HP ENVY Phoenix 860-081no (Intel Core i7-6700K CPU, NVIDIA GeForce GTX 980 TI GPU) desktop computer running Debian GNU/Linux as the operating system.

### Design

**Fig. 1:**
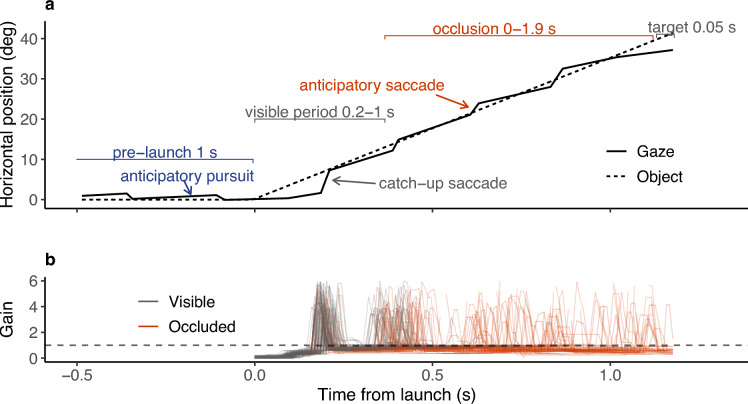
(**a**) Example trial (participant 7) from pre-launch to target reappearance, with dashed and solid lines showing object and gaze horizontal position. Anticipatory pursuits with return saccades are made before launch, pursuit with catch-up saccades during visually guided tracking, and pursuit with mainly anticipatory ’jump-ahead’ saccades during occlusion. (**b**) Gain (gaze velocity/target velocity; undefined pre-launch) within all trials of participant 7. Visually guided and occluded tracking are denoted by grey and orange lines, respectively. Unity gain (perfect velocity match) is marked with a dashed line. Catch-up saccades are observed as large spikes; pursuit gain is decaying over time.

Each participant tracked an object moving along a linear horizontal path, alternating direction, for a total of 120 trials in four blocks (a training block without occlusions, and three occlusion blocks). Each trial began with the object positioned at approximately 27 degrees to the left or right of the screen’s horizontal midpoint, visible for one second before launch. In occlusion trials, the moving object was visible for 0.2–1 seconds (randomized), occluded for 0–1.9 s (randomized; *Mdn* = 0.53 s, *M* = 0.58 s), and reappeared briefly with a Landolt C for 0.05 s (see Fig. 1, and task video at https://github.com/ttammi/trackquality). Participants reported the letter orientation (left, right, up, down) and received immediate visual feedback (correct/incorrect).

Randomized reappearance times encouraged gaze to stay close to the invisible object position at all times during occlusion, because participants could not know where in time or space the target would reappear. Target velocity remained constant within a trial but varied randomly between trials (22.5–45.0 degrees per second, uniform distribution). In occlusion trials, the target always disappeared before crossing the horizontal midpoint of the screen.

### Procedure

Participants were asked to track the moving (visible) target and to complete the discrimination task as accurately as possible. No instruction on occluded tracking or response speed was given. After initial setup and calibration of the eye tracker, participants completed the experiment at their own pace, free to take breaks between blocks or to withdraw from the experiment at any point. The eye tracker was re-calibrated between blocks. The full procedure took approximately 45 minutes to complete.

### Data processing and analysis

Based on confidence ratings of gaze data points by the Pupil Capture software (0–1; ratio of detected pupil edge length and fitted ellipse circumference), data with confidence less than 0.8 were excluded. Gaze positions were mapped as visual angles by assuming an 80$^{\circ }$ horizontal field of view for the monitor, therefore using a factor of 80/1920 to compute visual angles from monitor pixel positions. To account for head movement, head-to-screen position and orientation were estimated using an unscented Kalman smoother (see Tammi et al., 2022^12^ for details). Saccades and smooth pursuits were classified using the NSLR-HMM method^25^. It first estimates a piecewise linear regression of the gaze signal, after which the resulting linear segments are classified using a Hidden Markov Model based on the segments’ velocities and changes in direction.

All reported confidence intervals were computed using the percentile bootstrap method with 5000 resamples, using the R *boot* package^26^.

### Task accuracy model

To quantify the factors influencing perceptual performance, i.e. task accuracy, we formulated a psychometric model based on discrimination task outcomes (success or failure), displacement (gaze-target positional difference), and slippage (gaze-target velocity difference). Displacement and slippage were measured *at the time of target reappearance*, based on the assumption that this moment is most directly relevant to the participant’s response.

We assumed a psychophysical relationship between displacement, slippage, and visual perception: namely, that visual accuracy decays exponentially with increasing absolute displacement and slippage. We modelled task accuracy *A* for a task with absolute displacement *d* and absolute slippage *s* as:

$$\begin{aligned}A = \gamma + (1 - \gamma )(1 - \lambda ) F(d, s; \beta _d, \beta _s)\end{aligned}$$

where $\gamma$ is chance level (fixed at .25 for the four-alternative forced choice task), $\lambda$ is lapse probability, and *F* is a tracking quality function of the form

$$\begin{aligned}F(d, s; \beta _d, \beta _s) = exp(-(\beta _d d )^2)exp(-(\beta _s s )^2)\end{aligned}$$

with scaling coefficients $\beta _d$ and $\beta _s$ representing sensitivity to displacement and slippage, respectively. By definition, tracking with both zero-displacement and zero-slippage corresponds to discrimination task success rate of $1 - \lambda + \lambda \gamma$, plateauing at the chance level $\gamma$ (.25) as either *d* or *s* approaches large values.

As in Wichmann and Hill^27^, the lapse probability parameter $\lambda$ was used as a subject-specific constant, representing propensity for attentional lapses or random mistakes throughout the task. This assumption simplifies the model by ignoring potential temporal fluctuations in attention but captures participant-level differences in task engagement. Similarly, the scaling coefficients $\beta _d$ and $\beta _s$ were estimated separately for each participant.

We used absolute values of displacement and slippage, assuming that perceptual accuracy declines symmetrically with their increasing magnitude, irrespective of direction. Moreover, we treated displacement and slippage as independent factors, without including an interaction term, to avoid overfitting.

All three parameters ($\beta _d$, $\beta _s$, and $\lambda$) were estimated by maximum-likelihood using the DIRECT-L algorithm^28^ as implemented in the R *nloptr* package^29^.

#### Tracking quality

Based on the task accuracy model, we derived an integrated tracking quality measure to evaluate gaze behavior during occlusion, considering both displacement and slippage at a given time. For the tracking quality measure, we omitted the parameters related to the behavioral task structure, namely the chance level and lapse probability. This means that tracking quality could range from 0 to 1, with 1 implying ‘perfect-quality’ tracking.

Because the level of positional accuracy and velocity matching required to meet the task goals (succeed in the discrimination task) can vary between individuals, for example due to visual acuity differences, we used the participant-level model parameters in assessing tracking quality. Using the mean parameter values might, for example, underestimate the tracking quality of an individual with very good visual acuity, i.e. for whom perfect position matching is not necessary to meet the task goals.

Since participants were unaware of when the target would reappear during occlusion periods, we assume they aimed to maintain ‘good’ tracking—in both position and velocity—throughout the occlusion. Therefore, this measure offers a way to evaluate tracking quality continuously throughout the occlusion.

## Results

### Characterization of gaze behavior and task performance

Participants displayed strong performance in the discrimination task in occlusion trials, with nine out of ten achieving success rates between 69 % and 93 % (*M* = 78, 95% CI [73, 83]), against a random chance of 25 % (see Supplementary Table S1). This was, on average, ten percentage points worse than in the training block without occlusions (95% CI [5, 15]).

**Fig. 2:**
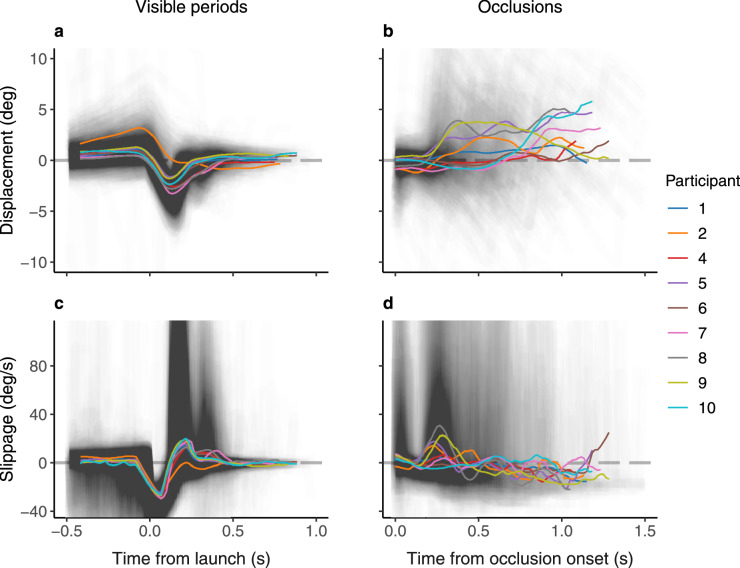
Time series of two measures of visual stabilization: gaze-target displacement (positional difference; (**a**,**b**)) and slippage (velocity difference; (**c**,**d**)), during visible periods (**a**,**c**) and occlusions (**b**,**d**). Lines show participant-wise means (smoothed with rolling mean, window width = 100 ms). Before launch, when the target was stationary, gaze was ahead of the target in both positional and velocity terms. For most participants, there was an initial lag when the target started moving, followed by at least one catch-up saccade. During occlusion, smooth pursuit speed remained below target speed but saccades were made, many of which landed substantially ahead of the target position, leading the gaze to be ahead on average.

Figure 1 illustrates typical gaze behavior observed within a single trial (selected based on qualitative visual inspection of gaze behavior across participants and trials), and Fig. 2 shows the temporal evolution of gaze-target displacement (positional difference) and slippage (velocity difference). Before the target started moving, the gaze position was, on average, slightly ahead of the target: participants made anticipatory pursuits to the upcoming motion direction, which were usually followed by return saccades back to the starting position. Shortly after launch, gaze tended to lag behind, requiring one or two saccades to catch up with the visible target. After the initial catch-up saccades, gaze-target displacement during the visible period was low and the target was tracked with smooth pursuit, accompanied by small catch-up saccades.

In contrast, during occlusion, smooth pursuit was accompanied by compensatory and anticipatory saccades. There was a decay in smooth pursuit: participant-wise median gaze speeds 600 ms into the occlusion were, on average, 62 % of the initial value (95% CI [52, 72]). This decay contributed to velocity slippage as well as positional displacement.

Moreover, we observed that the majority of saccades landed ahead of the object position. Participant-wise median landing displacements averaged 1.50 degrees (95% CI [0.88, 2.19]) during occluded tracking, compared to $-0.26$ degrees (95% CI [$-0.59$, 0.07]) during visually guided tracking. The mean paired difference in landing displacement between visible and occluded periods was 1.75 degrees (95% CI [1.21, 2.36]), indicating a change in the positional displacement distribution compared to visually guided tracking. The distributions of signed displacement and slippage during visible and occluded periods can be found in Supplementary Fig. S1.

### Displacement, slippage, and task accuracy

**Fig. 3:**
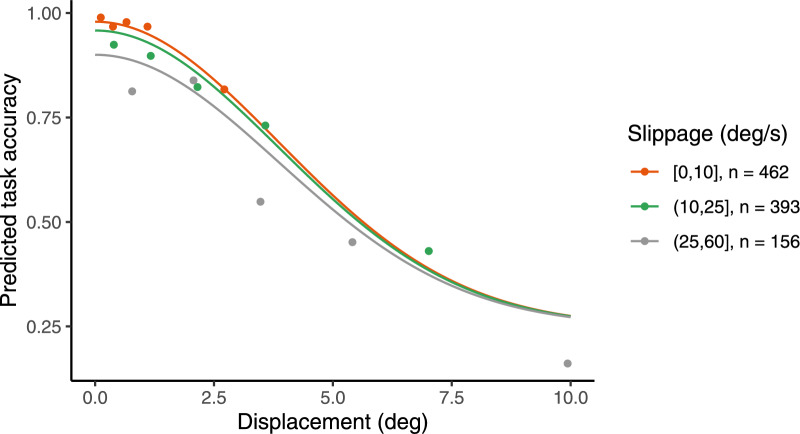
Observed overall success rates (points) per slippage (colors) and displacement (x-axis) bins. Task accuracy model predictions (lines) were computed using the mean values of participant-wise model parameters. The task accuracy model is based on the integrated tracking quality metric, which includes both displacement and slippage, as well as task-dependent chance level and lapse probability.

We found that tracking the target with close position and limited speed difference had a positive effect on discrimination task performance. Across participants, observed task accuracy was highest with small displacement, exceeding 90 percent when displacement was at most 2.5 degrees. Even with great slippage values, overall task accuracy remained high as long as displacement was small, highlighting displacement as the dominant factor in task success. Likewise, as displacement increased, success rates dropped sharply, diminishing the influence of slippage in high-displacement conditions.

**Fig. 4:**
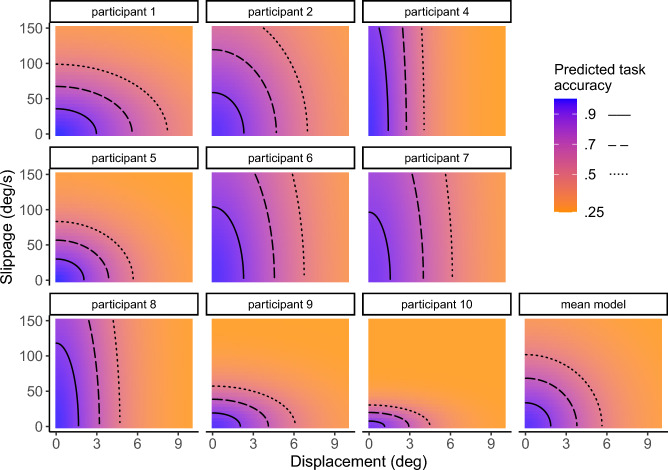
Predicted task accuracy for different combinations of displacement and slippage, shown separately for each participant (9 panels) and for the model using mean parameters across participants (bottom right). Background color and contour lines indicate the predicted probability of correct discrimination response based on the two tracking quality dimensions. Across participants, displacement showed a consistent effect on task accuracy, while the influence of slippage varied more.

Correspondingly, the role of displacement was emphasized by the task accuracy model; Fig. 3 shows the observed task accuracy rates and mean model predictions for given displacement and slippage values. Means of the participant-wise model parameters were $\beta _d$ = 0.18 (95% CI [0.16, 0.21]), $\beta _s$ = 0.01 (95% CI [0.005, 0.02]), and $\lambda$ = 0.03 (95% CI [0.01, 0.05]). The effect of positional displacement was consistent across participants (see Supplementary Table S2). In contrast, the effect of slippage varied more and was generally modest in comparison to displacement (see Fig. 4 for a participant-wise figure of these effects).

Model comparisons based on both log-likelihood and AIC, summed across participant-wise models, are reported in Table 1. Removing the displacement parameter ($\beta _d$) substantially worsened model fit, whereas removing the slippage parameter ($\beta _s$) had a smaller impact. Moreover, the reduced model without lapse rate ($\lambda$) attained the lowest AIC. Nevertheless, we suggest retaining the lapse rate based on its theoretical relevance and to account for cross-participant variation.

**Table 1:** Summed log-likelihoods and AIC values across participants for the full model (with free parameters $\beta _d$, $\beta _s$, and $\lambda$) and reduced models, from lowest to highest AIC.

Model	Log-likelihood	AIC
Remove $\lambda$	$-377.24$	790.48
Full model	$-369.88$	793.76
Remove $\beta _s$	$-383.29$	802.57
Remove $\beta _s$, $\lambda$	$-417.80$	853.60
Remove $\beta _d$	$-456.57$	949.15
Remove $\beta _d$, $\lambda$	$-481.78$	981.56
Remove $\beta _d$, $\beta _s$	$-511.19$	1040.38

### Occluded tracking quality

**Fig. 5:**
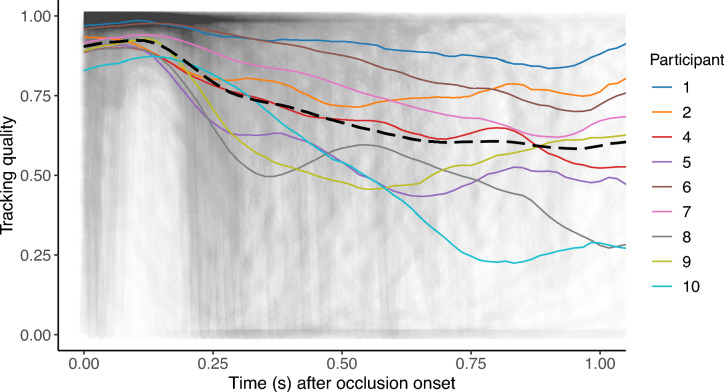
Time series of tracking quality over the course of occlusion. Colored lines denote participant-wise means per time point (smoothed with rolling mean, window width = 100 ms). Dashed black line shows the tracking quality based on the mean parameter model. After the first 150 ms, i.e. the latency of reacting to the disappearance of the target, tracking quality started to decay, reaching a plateau at around 600 ms.

Figure 5 shows the development of tracking quality—with range of possible values between zero and one—over occluded time. On average, the tracking quality value was .89 (95% CI [0.86, 0.92]) at the beginning of occlusion. Tracking quality decayed over time; after 600 ms it was, on average, .63 (95% CI [0.52, 0.74]). In contrast, tracking quality during visually guided tracking remained high (*M* = 0.97, 95% CI [0.96, 0.98], at 600 ms after launch).

As our task accuracy model suggests, good-quality tracking during occlusion was predominantly marked by maintaining a small enough distance from target position, achieved by smooth pursuit and small jump-ahead saccades. Pursuit decay, if unaccompanied by saccades, resulted in both increased slippage and displacement, thereby impairing tracking quality. Furthermore, large-amplitude saccades made shortly after occlusion onset were reflected as a drop in tracking quality, due predominantly to the large displacement created but also to the slippage during saccade execution (see Fig. 2).

## Discussion

In this study, we characterize the patterns of gaze behavior observed when participants tracked linear motion through intermittent occlusions to perform a visual discrimination task. We thus aim to understand the implications of gaze patterns for task performance. We introduce a model to understand how gaze behavior relates to task goals, focusing on the relationship between displacement (gaze-target positional difference), slippage (gaze-target velocity difference), and discrimination task performance. With this model, we provide a metric for objectively assessing how well tracking is maintained during occlusion periods.

Our detailed characterization of gaze behavior revealed distinct patterns throughout the tracking task. Before motion onset, participants usually exhibited anticipatory smooth pursuits with return saccades back to the stationary target position. As the launch moment approached, gaze typically led the stationary target slightly, reflecting anticipation of the object’s future trajectory, consistent with previous findings on anticipatory eye movements (see e.g. Santos & Kowler, 2017^30^). During the period of visible motion and after the initial catch-up saccades, participants primarily used smooth pursuit to track the moving object, which was effective for the perceptual task. In contrast, during occlusion, pursuit speed decayed below target speed, necessitating saccades to maintain close tracking of the object. Notably, gaze often led the target during occlusion, due to saccades landing ahead of the object position. Our task design, including random occlusion durations, prevented participants from anticipating the exact reappearance timing or location based on prior experience. This encouraged readiness for target reappearance throughout the occlusion period. Our design is different from, for example, prediction motion tasks where participants are asked to estimate the arrival time of a visual target at a fixed location^6^.

To quantify our observations of occluded tracking behavior, we developed a psychometric model that incorporates both displacement and slippage as key factors influencing visual target perception. Displacement reflects the positional difference between gaze and target, arising from both smooth pursuit drift and jump-ahead saccades made during tracking, with catch-up saccades reducing this difference. Slippage, representing the velocity difference between gaze and target, is mostly driven by a gradual decay in smooth pursuit velocity over time, although saccades can also influence this measure.

Importantly, discrimination performance relies on maintaining a small enough positional displacement to resolve target details without crowding, while sufficiently matching target velocity to avoid blurring. This relationship was evident in the tracking quality metric during occlusion, which decreased with decaying pursuit gain, reflecting the increased difficulty in maintaining positional accuracy without a visual stimulus. In fact, positional accuracy was generally more crucial to task performance than velocity matching, suggesting that the target could in some cases be perceived correctly even with large velocity differences: for example, a saccade being made at the moment of target reappearance. While saccadic suppression generally leads to impairments in visual perception^31–33^, some research suggests that it is possible to acquire visual information during saccades, although in task designs different from ours^34,35^. Moreover, the possible advantage of the ’jump-ahead’ saccades observed during occlusion, compared to catch-up saccades, could be related to maintaining a small enough positional displacement for a longer time, reducing the need for frequent saccades and thereby facilitating visual perception. Our findings bear similarity to those of Palidis and colleagues^11^, who studied fully visible linear trajectories and found that both smooth pursuit tracking and position error minimization by saccades were advantageous for dynamic visual acuity performance.

Our tracking quality metric, derived from the task accuracy model, provides a tool for continuous evaluation of tracking performance throughout occlusion periods. While discrimination task outcome at target reappearance provides a snapshot of tracking performance, the tracking quality metric gives a fuller picture, allowing for a more thorough examination of tracking strategies and the temporal evolution of gaze dynamics within occlusions. Furthermore, we include the full gaze signal in our model, not explicitly differentiating between smooth pursuit and saccadic eye movements. While previous studies on occluded object tracking have often focused on a specific type of eye movement, typically de-saccaded smooth pursuit^1,2,36^, our approach offers a more comprehensive account of tracking behavior, taking into consideration the interplay between smooth pursuits and saccades in object tracking^8^ (see also Adams et al., 2015^37^).

In our model, we assume that perceptual accuracy decreases symmetrically with increasing displacement or slippage, regardless of direction. While this assumption simplifies the model, it also omits potential asymmetries in gaze behavior. For example, slippage was most often negative, typically resulting from smooth pursuit or fixation below target speed. Likewise, negative displacement was usually linked to the positional difference created by smooth pursuit decay while positive displacement resulted from anticipatory saccades landing ahead of the target. While we use absolute values for simplicity, future studies could explore the possible asymmetries in tracking behavior in more detail.

The lapse rate was modelled as a constant, subject-specific parameter. This reflects the common assumption that attentional lapses are mostly participant-dependent but stable throughout the task^27^. While this assumption simplifies the model, it may overlook temporal fluctuations in attention and introduce slight biases in parameter estimates^38^. Here, although a reduced model without the lapse rate marginally outperformed the full model fit by AIC, we retained the lapse rate for theoretical consistency. In the future, alternative approaches could be explored to address attentional fluctuations in this context, possibly even using measures such as reaction times in detecting attentional lapses.

While our sample size was small, it was typical for eye-tracking studies of occluded target tracking^1,2,4,20,39^. With a high number of trials per participant, it was sufficient for demonstrating the tracking quality concept using a within-participant modelling approach, offering a stepping stone for future research. However, the small number of participants constrained our ability to systematically investigate individual differences. Future research with larger samples could study whether between-participant variations in gaze patterns – for example, reliance on saccades versus smooth pursuit during occlusion – might reflect differences in strategy, learning history, or oculomotor control, among other factors.

Our task reflects some features of scenarios commonly encountered in everyday life, such as observing moving vehicles or objects across a landscape, which may temporarily disappear from view or be obscured. However, our task characteristics do not capture the richness of motion observed in natural environments, as real-world motion often involves nonlinear trajectories, acceleration, or interaction with other objects. For example, in a more complex or unpredictable trajectory, the trade-offs between matching target velocity and maintaining positional accuracy may be emphasized: a predominantly saccadic strategy may be necessary in maintaining positional accuracy along nonlinear trajectories^12^. Furthermore, our task design, incorporating a discrimination task and immediate feedback, may introduce strategies not typically engaged during naturalistic motion tracking.

On the other hand, this emphasizes the need for an objective performance metric when evaluating visual tracking: the principle of building a tracking quality measure based on task goals and conditions, rather than our model per se, could be generalized to various scenarios. In future studies, the same principle could be applied to more complex designs with different elements affecting performance. The key prerequisites are that participants have clear goals and feedback, and that the achievement of task goals can be measured. One interesting possibility would also be to manipulate and contrast different types of task goals and stimuli while keeping other factors constant.

In addition, it would be valuable to investigate how gaze behavior and tracking quality develop over repeated trials, assuming that participants adapt their gaze behavior based on feedback and experience^23^. Understanding temporal changes in tracking quality could provide insights into the evolution of internal estimates of target motion characteristics. This could also shed light on individual variation in the development of anticipatory gaze behavior and gaze strategies to enhance task performance.

All in all, our study complements previous approaches to gaze behavior in occluded tracking tasks, which have often focused on isolated gaze components. By examining the full gaze signal and anchoring our analysis to task performance, we highlight how gaze strategies may be influenced by specific task goals and conditions. We provide new insights into anticipatory visual tracking, enhancing the understanding of what constitutes good-quality tracking and how it can be operationalized.

## Supplementary Information

Below is the link to the electronic supplementary material.

Supplementary Material 1

## Data Availability

The datasets of the current study are available in the GitHub repository, https://github.com/ttammi/trackquality.

## Code availability

Code for analysis and modelling is available in the GitHub repository, https://github.com/ttammi/trackquality.
