# Supplementary material for: Quantifying tracking quality during occlusion with an integrated gaze metric anchored to task performance
Source: Sci Rep. 2025 Aug 29;15:31858. doi: 10.1038/s41598-025-17519-8 (PMC12397211; doi:10.1038/s41598-025-17519-8)
Supplement: Supplementary file 1 — Supplementary Material 1 [file 41598_2025_17519_MOESM1_ESM.pdf]

SUPPLEMENTARY INFORMATION: Quantifying Tracking Quality During Occlusion With an Integrated Gaze Metric Anchored to Task Performance

Tuisku Tammi[1,*], Jami Pekkanen[1], Benjamin Ultan Cowley[2], and Otto Lappi[1]

[1]Cognitive Science, University of Helsinki, Helsinki, Finland
[2]Faculty of Educational Sciences, University of Helsinki, Helsinki, Finland
[*]tuisku.tammi@helsinki.fi

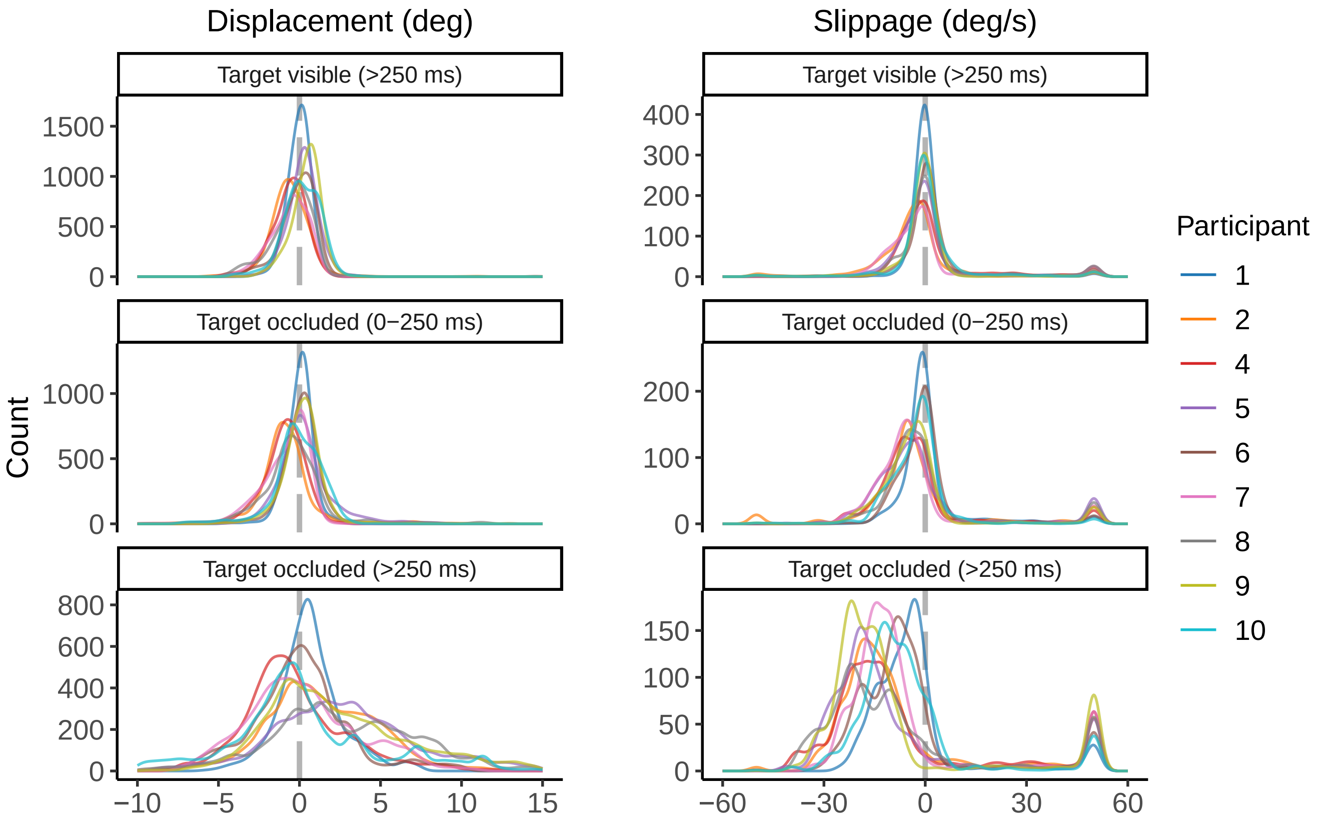

Figure S1. Distributions of gaze-target positional displacement and slippage during visually guided tracking (after the initial catch-up phase, i.e. at least 250 ms after launch), and during the initial stage of occlusion (0-250 ms), and the rest of occluded tracking (after 250 ms). Note that all slippage values of at least 50 deg/s have been collapsed to 50 for illustration purposes.

Participant	Success rate		Displacement		Slippage	
	Visible	Occluded	Visible	Occluded	Visible	Occluded
	%	%	M (SD)	M (SD)	M (SD)	M (SD)
1	100.0	93.3	-0.29 (0.74)	0.66 (0.56)	-1.68 (14.42)	-2.60 (6.53)
2	93.3	83.3	-0.21 (0.93)	0.94 (1.23)	-5.79 (10.59)	-1.47 (13.46)
4	90.0	75.3	-0.89 (1.00)	-0.07 (0.78)	-1.86 (15.42)	-2.35 (8.67)
5	90.0	72.2	-0.12 (0.81)	2.68 (1.56)	-1.75 (15.26)	-3.05 (14.63)
6	86.2	86.7	-0.24 (1.06)	0.24 (0.69)	-0.81 (17.05)	-0.75 (11.43)
7	83.3	80.9	-0.89 (1.39)	0.58 (1.76)	-0.85 (22.17)	-0.66 (9.82)
8	80.0	73.0	-0.61 (1.27)	2.52 (1.90)	-0.03 (15.95)	-2.14 (17.39)
9	93.3	69.0	0.05 (0.93)	1.93 (1.45)	-1.58 (17.20)	-4.79 (13.47)
10	73.3	69.7	-0.22 (1.06)	1.33 (2.35)	-0.67 (15.84)	-1.08 (7.46)

Table S1. Discrimination task success rates per participant in visible-only (each n = 30) and occlusion (each n = 90) trials, and mean (SD) displacement and slippage during visible and occluded tracking. Discrimination task chance level = 25 %.

	Participant									***M***
	1	2	4	5	6	7	8	9	10	
Parameter										
β_d	0.13	0.15	0.26	0.18	0.15	0.16	0.22	0.17	0.22	0.18
β_s	0.011	0.006	0.002	0.013	0.003	0.003	0.003	0.018	0.033	0.010
λ	$< .001$	.026	.010	$< .001$	.019	.074	.009	.022	.074	.026
Log-likelihood	-15.74	-36.62	-44.65	-40.02	-33.19	-47.53	-47.69	-44.16	-60.28	-41.10

Table S2. Participant-wise and averaged parameters and log-likelihood values for the task accuracy model: β_d = displacement scaling coefficient, β_s = slippage scaling coefficient, λ = lapse probability.
